# Managing the Difficult Airway in Adults with Life-Threatening Cervical Infections: A Scoping Review

**DOI:** 10.3390/healthcare14172728

**Published:** 2026-08-26

**Authors:** Andrea Migliorelli, Marianna Manuelli, Andrea Bianchino, Giovanni Cammaroto, Andrea Ciorba, Francesco Stomeo, Stefano Pelucchi, Chiara Bianchini

**Affiliations:** ENT & Audiology Unit, Department of Neurosciences, University Hospital of Ferrara, 44100 Ferrara, Italy

**Keywords:** deep neck infections, epiglottitis, supraglottitis, difficult airway, tracheostomy, airway assessment, airway intervention

## Abstract

**Background:** Airway management in patients with cervical infections remains a major challenge because progressive swelling and distortion of the upper airway may rapidly lead to life-threatening airway obstruction. Despite advances in imaging and advanced intubation techniques, the optimal management strategy remains controversial. This scoping review aimed to evaluate the current evidence on airway assessment and management of adult patients with cervical infections. **Methods:** A scoping review was conducted according to the PRISMA-ScR recommendations. PubMed/MEDLINE, Scopus, and Embase were systematically searched for English-language studies published between January 2006 and May 2026. Studies evaluating airway management in adult patients with deep neck infections or acute infectious epiglottitis/supraglottitis were included. Data regarding study characteristics, airway management strategies, predictors of airway intervention, and clinical outcomes were extracted and synthesized descriptively. **Results:** Fourteen studies involving 3297 patients met the inclusion criteria. Endotracheal intubation was the most frequently reported airway management strategy, while tracheostomy remained essential in selected patients with extensive multispace infections, descending necrotizing mediastinitis, severe airway alteration, or anticipated prolonged airway protection. Clinical signs of respiratory compromise, multispace cervical involvement, mediastinal extension, and significant supraglottic edema were the most consistently reported predictors of airway intervention. Across the included studies, airway management strategies varied considerably according to disease severity, anatomical findings, institutional protocols, and operator experience. **Conclusions:** Current evidence suggests an individualized, multidisciplinary approach to airway management in adults with life-threatening cervical infections. Timely airway assessment integrating clinical evaluation, flexible endoscopy, and contrast-enhanced computed tomography is essential to guide appropriate airway intervention.

## 1. Introduction

Deep neck infections (DNIs) and acute infectious supraglottitis represent potentially life-threatening conditions because of their ability to rapidly compromise the upper airway [1,2]. Despite advances in antimicrobial therapy, diagnostic imaging, and surgical techniques, these infections continue to be associated with significant morbidity and, when airway compromise is not promptly recognized, potentially fatal outcomes [3,4,5]. Progressive inflammatory edema, abscess formation, trismus, and distortion of physiological cervical anatomy may rapidly transform an initially stable airway into a difficult or impossible airway, making early recognition and timely intervention essential [6,7].

Airway management remains a key component of treatment in patients with cervical infections [8]. However, determining whether, when, and how to secure the airway remains one of the most challenging aspects of clinical management.

While contemporary airway management offers multiple options for securing the airway, the optimal strategy remains controversial and should be individualized according to clinical and anatomical findings.

Although numerous retrospective studies have investigated airway management in specific clinical scenarios, including odontogenic infections, Ludwig’s angina, parapharyngeal and retropharyngeal abscesses, descending necrotizing mediastinitis, and acute infectious epiglottitis, the available evidence remains heterogeneous [6,9,10]. Most published studies focus on individual diseases or institutional experiences, while comprehensive reviews specifically addressing airway management across the spectrum of cervical infections are rare [5,11]. Furthermore, advances in airway devices, imaging modalities, and predictive models have introduced additional tools for airway assessment and management, although their specific impact in patients with cervical infections remains incompletely characterized.

The present scoping review provides a comprehensive overview of airway management across the spectrum of life-threatening cervical infections, integrating evidence from deep neck infections and acute infectious epiglottitis/supraglottitis. In addition, it summarizes the reported predictors of airway intervention and identifies current gaps in knowledge to guide future research.

Accordingly, the aim of this scoping review was to evaluate current evidence on airway management in adult patients with cervical infections, with a particular focus on deep neck infections and acute infectious epiglottitis/supraglottitis. Specifically, available evidence on airway assessment, indications for endotracheal intubation and tracheostomy, predictors of airway intervention, and the clinical implications of current management strategies was reviewed, while areas requiring further research were highlighted.

## 2. Materials and Methods

**Figure 1:**
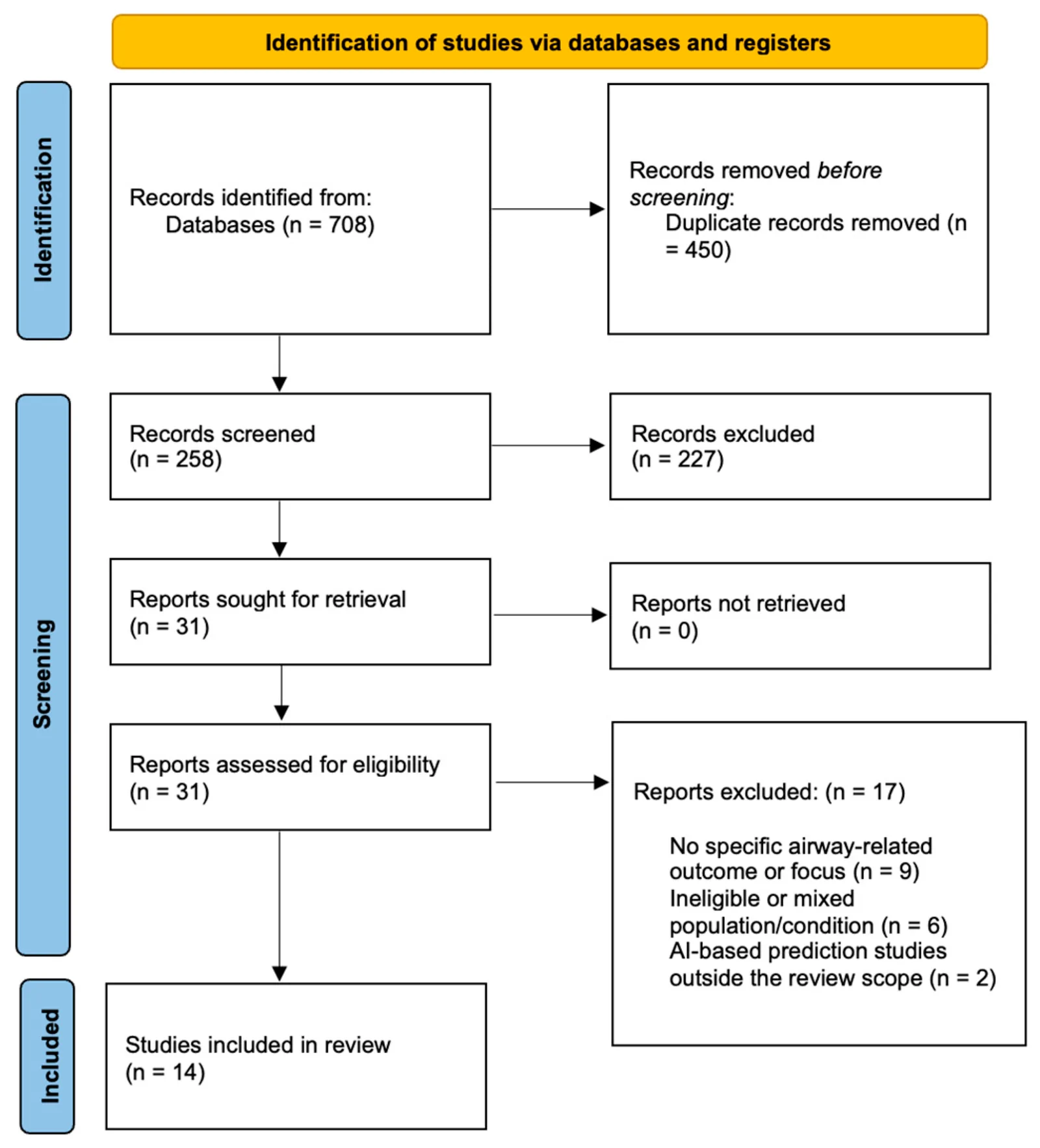
PRISMA-ScR flow diagram illustrating the study selection process.

A scoping review of the English-language literature investigating airway management in patients with DNIs and acute infectious epiglottitis/supraglottitis was performed using the PubMed/MEDLINE, Scopus, and Embase databases. The literature search was conducted from January 2006 to May 2026 and followed the Preferred Reporting Items for Systematic Reviews and Meta-Analyses extension for Scoping Reviews (PRISMA-ScR) recommendations [12] (Figure 1). The search period was selected to capture contemporary evidence on airway management strategies and diagnostic assessment in patients with cervical infections.

The search strategy combined terms related to deep neck infections and supraglottitis with terms related to airway management. The PubMed/MEDLINE search strategy was as follows: (“Epiglottitis”[Mesh] OR epiglottitis[tiab] OR supraglottitis[tiab] OR “Neck Abscess”[Mesh] OR “deep neck infection*”[tiab] OR “retropharyngeal abscess”[tiab] OR “parapharyngeal abscess”[tiab] OR “Ludwig’s angina”[tiab]) AND (“Tracheostomy”[Mesh] OR tracheostom*[tiab] OR tracheotom*[tiab] OR “surgical airway”[tiab]). The search syntax was adapted according to the requirements of Scopus and Embase. The last search was performed on 18 May 2026.

The search strategy was designed primarily to identify studies addressing airway intervention in the setting of cervical infections rather than to systematically evaluate individual intubation devices or techniques. Consequently, device- and technique-specific terms such as videolaryngoscopy, GlideScope, C-MAC, awake intubation, and flexible bronchoscopic intubation were not included as independent search terms.

Studies were considered eligible if they met the following inclusion criteria: (i) original full-text articles published in English; (ii) studies evaluating patients with deep neck infections or acute infectious epiglottitis/supraglottitis in the adult population; (iii) studies investigating airway management strategies, including endotracheal intubation, prolonged postoperative intubation, tracheostomy, or surgical airway; and (iv) studies reporting airway-related outcomes, predictors of airway intervention, or comparisons between different airway management strategies. Case reports, conference abstracts, editorials, letters, narrative reviews, studies exclusively involving pediatric patients, studies without specific airway-related outcomes, and duplicate or overlapping patient cohorts were excluded.

The initial database search identified 708 records. After duplicate removal, 258 articles remained for title and abstract screening. Following preliminary evaluation, 31 studies underwent full-text assessment for eligibility. At the end of the selection process, 14 studies fulfilled all inclusion criteria and were included in the present scoping review [6,7,9,10,13,14,15,16,17,18,19,20,21,22].

Two investigators (A.M. and M.M.) independently screened titles, abstracts, and full texts according to the predefined eligibility criteria. Disagreements regarding study inclusion were resolved through discussion with a senior reviewer (C.B.) until consensus was reached. Data were then independently extracted using a standardized data-charting form by the two reviewers.

Data extracted from the selected studies included country of origin, study design, study period, sample size, type of infection, airway management strategy, indications for airway intervention, predictors of intubation or tracheostomy, postoperative airway management, and principal clinical outcomes. Given the expected heterogeneity in study design, patient populations, airway management strategies, and reported outcomes, a quantitative meta-analysis was not performed. Therefore, findings were synthesized descriptively and narratively. In accordance with the PRISMA-ScR methodology, no formal risk-of-bias assessment was undertaken. The protocol for this review was registered on the Open Science Framework (OSF) Registries with the Registration: https://osf.io/7e4y5 (accessed on 28 June 2026).

## 3. Results

### 3.1. Study Characteristics

The included studies were published between 2011 and 2026 and comprised a total of 3297 adult patients with DNIs, acute infectious epiglottitis/supraglottitis, or descending necrotizing mediastinitis (including 1882 patients from the nationwide administrative database study by Konishi et al. [19]). Although the literature search covered studies published from January 2006 onwards, the earliest study meeting the inclusion criteria was published in 2011. Thirteen studies were retrospective observational cohorts, whereas only one combined prospective and retrospective data collection [21]. The studies originated from Europe, North America, and Asia, reflecting the international interest in the management of infectious airway emergencies.

Nine studies investigated airway management in deep neck infections, including odontogenic infections, Ludwig’s angina, parapharyngeal and retropharyngeal abscesses, cervicofacial cellulitis, and descending necrotizing mediastinitis [6,7,10,13,16,17,19,20,21], whereas five studies focused on acute infectious epiglottitis or supraglottitis [9,14,15,18,22].

Despite differences in study design, patient populations, and disease severity, all included articles addressed airway management as a primary or major secondary outcome. The principal objectives included comparison of airway management strategies, identification of predictors of airway intervention or tracheostomy, evaluation of postoperative airway management, and characterization of clinical outcomes associated with different airway approaches. The main characteristics of the included studies are summarized in Table 1 and Table 2.

### 3.2. Airway Management in Deep Neck Infections

Nine of the included studies evaluated airway management in adult patients with deep neck infections, including odontogenic infections, Ludwig’s angina, parapharyngeal and retropharyngeal abscesses, cervicofacial cellulitis, and descending necrotizing mediastinitis [6,7,10,13,16,17,19,20,21]. Despite differences in patient populations and disease severity, all studies assessed airway management either as the primary outcome or as a major component of the overall treatment strategy.

Endotracheal intubation was the most frequently reported airway management technique across the included studies. In the retrospective series by Tapiovaara et al. [6], involving 202 surgically treated patients, endotracheal intubation represented the preferred initial airway management strategy (82% of patients), whereas primary tracheostomy was performed in 35/202 patients (17%), with secondary tracheostomy required in a further 25/202 (12%) patients.

Immediate postoperative extubation was achieved in approximately half of the cohort, while the remaining patients required prolonged postoperative airway support or admission to the intensive care unit. Similarly, Wolfe et al. [7] reported successful airway management in all patients using advanced airway techniques, including awake fiberoptic intubation, videolaryngoscopy, and retrograde intubation, without the need for surgical airway.

Tracheostomy was consistently reserved for patients presenting with more advanced disease or anticipated difficult airway management. In the study by Chen et al. [17], tracheostomy was required in 44 of 403 patients (10.9%). Independent predictors associated with tracheostomy included age ≥ 65 years, involvement of three or more deep neck spaces, and descending mediastinitis. Likewise, Gehrke et al. [16] reported mediastinal involvement in 45/218 patients, with tracheostomy performed in 82.2% of patients with mediastinal involvement compared with 3.4% of those without mediastinal involvement.

The anatomical extent of infection represented another important determinant of airway management. Garcia et al. [13] reported that retropharyngeal infections were associated with more severe disease, whereas parapharyngeal involvement was associated with a more complicated clinical course requiring additional airway support. Similar findings were observed in the nationwide Japanese database study by Konishi et al. [19], in which patients with parapharyngeal abscesses underwent tracheostomy and intensive care admission more frequently than those with retropharyngeal abscesses.

Descending necrotizing mediastinitis was evaluated separately by Kim et al. [10]. All patients underwent emergency surgical drainage followed by postoperative airway management. Short-term postoperative endotracheal intubation was successfully employed in selected patients, whereas tracheostomy was reserved for patients requiring prolonged airway protection or in whom endotracheal airway management was considered unsuitable.

More recently, Iwata et al. [20] investigated postoperative airway management in patients with severe odontogenic deep neck infections. Seven patients required prolonged postoperative airway support or tracheostomy. Arytenoid edema, laryngeal edema, and para- or retropharyngeal abscesses were significantly associated with difficult postoperative airway management. Similarly, Hennocq et al. [21] evaluated patients with cervicofacial cellulitis and found that dysphagia and heterogeneous cervical collections were associated with tracheostomy, although this analysis incorporated a retrospective cohort selected on the basis of having undergone tracheostomy. In contrast, mouth opening increased after induction in all patients with available paired measurements (17/17), suggesting that trismus alone may not be a reliable predictor of difficult airway management after induction of general anesthesia.

Overall, the included studies reported considerable variability in airway management strategies, reflecting differences in disease severity, anatomical extension, and institutional practice. Endotracheal intubation represented the most frequently adopted initial airway management technique, whereas tracheostomy was generally performed in patients with extensive cervical infection, mediastinal extension, difficult airway anatomy, or the need for prolonged postoperative airway protection.

### 3.3. Airway Management in Acute Infectious Epiglottitis and Supraglottitis

Five studies specifically evaluated airway management in adult patients with acute infectious epiglottitis or supraglottitis [9,14,15,18,22]. Although upper airway obstruction remains the most feared complication of these conditions, the need for advanced airway intervention varied according to the clinical setting and patient selection. In unselected cohorts, the majority of adult patients were successfully managed without advanced airway intervention [9,18], whereas higher intervention rates were reported in selected populations [14,15]. Conservative treatment, including close clinical observation, intravenous antibiotics, corticosteroid therapy, and serial endoscopic examinations, represented the most common therapeutic approach in patients not requiring airway intervention, while airway intervention was reserved for patients presenting with signs of impending airway compromise.

Among patients requiring airway protection, both endotracheal intubation and tracheostomy were used to secure the airway. Tapiovaara et al. [14] specifically evaluated 42 adults with acute epiglottitis or supraglottitis who underwent airway intervention: 21/42 (50%) were managed with endotracheal intubation and 21/42 (50%) with tracheostomy. Tracheostomy was associated with lower treatment costs, while procedure-related complications occurred in 3/21 intubated patients and 8/21 tracheostomized patients, although this difference was not statistically significant.

Shaikh et al. [15] evaluated 118 patients with acute adult supraglottitis admitted to the surgical intensive care unit. Endotracheal intubation was required in 54/118 patients (45.8%), intubation was unsuccessful in 12/118 (10.2%), and tracheostomy was performed in 15/118 (12.7%). These findings highlight the substantial need for airway intervention in this selected critically ill population.

Felton et al. [18] reported that advanced airway management was required in a minority of patients with acute epiglottitis. Airway interventions included endotracheal intubation, tracheostomy, and emergency cricothyrotomy. Severe adverse events, including anoxic brain injury and death, were reported as complications following difficult airway management.

Likewise, Penella et al. [9] reported that airway intervention was performed in approximately 17% of adult patients with infectious supraglottitis. Endotracheal intubation represented the most common intervention, followed by tracheostomy. Patients managed conservatively generally experienced an uncomplicated clinical course and did not require escalation of airway support during hospitalization.

Pineau et al. [22] evaluated 28 adults with acute epiglottitis, of whom 10 required intubation. On multivariate analysis, dyspnea and supraglottic extension of edema were the main factors associated with the decision to intubate, highlighting the importance of combining clinical presentation with endoscopic assessment.

Overall, the reviewed studies demonstrated that advanced airway intervention was necessary in only a minority of adults with acute infectious epiglottitis or supraglottitis. When airway protection became necessary, endotracheal intubation represented the most frequently adopted strategy, whereas tracheostomy was reserved for selected patients in whom prolonged airway protection or difficult airway anatomy precluded safe endotracheal management.

### 3.4. Predictors of Airway Intervention

Several studies investigated factors associated with the need for advanced airway management, identifying a combination of clinical, radiological, endoscopic, and patient-related variables that were consistently associated with airway intervention. Although the reported predictors varied among studies, a common finding was that no single parameter was sufficient to determine the optimal airway strategy, emphasizing the importance of comprehensive patient assessment.

Clinical presentation represented the first determinant of airway management. In patients with acute infectious epiglottitis or supraglottitis, stridor, dyspnea, voice changes, dysphagia, and sialorrhea were the most frequently reported indicators of airway alteration. Felton et al. [18] identified stridor, dyspnea, and voice changes as the clinical variables most strongly associated with advanced airway intervention. Similarly, Penella et al. [9] reported that sialorrhea and the presence of an epiglottic abscess were significantly associated with the need for endotracheal intubation or tracheostomy. Pineau et al. [22] found that dyspnea and supraglottic extension of edema were the main factors associated with the decision to intubate in adults with acute epiglottitis. In patients with cervicofacial cellulitis, Hennocq et al. [21] also identified dysphagia as a significant predictor of tracheostomy.

Radiological findings represented another important component of airway assessment. Chen et al. [17] demonstrated that involvement of three or more deep neck spaces and descending mediastinitis were independent predictors of tracheostomy. Similar observations were reported by Garcia et al. [13], who described a more severe clinical course in patients with retropharyngeal and parapharyngeal infections. Likewise, the nationwide study by Konishi et al. [19] found that parapharyngeal abscesses were associated with a higher frequency of tracheostomy and intensive care admission than retropharyngeal abscesses.

Endoscopic findings also contributed to postoperative airway decision-making. Iwata et al. [20] reported that arytenoid edema and laryngeal edema were significantly associated with prolonged postoperative airway support or tracheostomy following surgical drainage of severe odontogenic deep neck infections. In contrast, Hennocq et al. [21] observed increased mouth opening after induction in all patients with available paired measurements (17/17), suggesting that trismus alone may not reliably predict difficult airway management and should be interpreted together with other clinical and radiological findings rather than considered an isolated indication for surgical airway management.

Patient-related characteristics were less consistently reported across studies. Increasing age, particularly age ≥ 65 years, was identified by Chen et al. [17] as an independent predictor of tracheostomy in patients with DNI. Conversely, Penella et al. [9] found that younger patients with infectious supraglottitis were significantly more likely to require airway intervention (*p* < 0.01) and also reported an association between active smoking and the need for airway intervention. These apparently contrasting age-related findings may reflect differences in the underlying patient populations and clinical conditions (DNI versus supraglottitis).

Overall, the reviewed literature demonstrated that airway intervention was more frequently required in patients presenting with extensive cervical infection, multispace involvement, mediastinal extension, significant supraglottic edema, epiglottic abscess, or marked respiratory compromise. The available studies consistently supported a multimodal airway assessment integrating clinical examination, flexible endoscopy, and contrast-enhanced computed tomography to identify patients at increased risk of airway deterioration.

The principal predictors of airway intervention identified across the included studies are summarized in Table 3.

## 4. Discussion

Based on the evidence identified in the present literature review, several issues warrant discussion.

### 4.1. Airway Assessment

The first and most important step in the management of patients with deep neck infections is an accurate assessment of the airway [6,7]. Unlike other causes of difficult airway, cervical infections are characterized by a dynamic process in which airway conditions may deteriorate rapidly because of progressive edema, extension of the inflammatory process, or abscess formation. Consequently, airway evaluation should not be considered a single event at presentation but rather a continuous process throughout the patient’s clinical course [6,7].

The studies included in this review consistently demonstrated that no isolated clinical finding is sufficient to guide airway management. Instead, the decision to secure the airway should be based on the integration of clinical examination, endoscopic assessment, and radiological imaging [9,16,18]. Clinical symptoms such as dyspnea, stridor, dysphagia, inability to tolerate secretions, muffled voice, and rapidly progressive neck swelling should immediately raise concern for impending airway compromise [9,16,18]. However, these findings should always be interpreted within the overall clinical context, as patients with apparently mild symptoms may still present with extensive deep neck involvement.

Flexible nasolaryngoscopy represents an essential component of the initial evaluation [5,6,15,18,22]. It allows direct visualization of supraglottic structures, assessment of airway patency, identification of laryngeal edema, and evaluation of secretion management without delaying treatment. Endoscopic findings may contribute to airway management decisions, particularly by assessing the extent of supraglottic edema and the need for airway intervention [22]. In recent years, several authors have emphasized the value of endoscopic examination during postoperative follow-up, particularly in patients managed with prolonged endotracheal intubation [9,10].

Contrast-enhanced computed tomography is equally important because it provides detailed information regarding the anatomical extent of infection, identifies the involvement of multiple deep neck spaces, and detects complications such as descending mediastinitis [10,16]. Several studies included in this review demonstrated that multispace infections, parapharyngeal or retropharyngeal involvement, and mediastinal extension are frequently associated with more complex airway management and a higher likelihood of airway intervention. Imaging findings should therefore be interpreted together with clinical and endoscopic evaluation when planning airway management [10,16,17].

Another recurring concept across the available literature is the importance of repeated airway assessment. Patients may initially appear clinically stable but subsequently deteriorate because of progression of edema or abscess formation, particularly during the first hours after hospital admission. For this reason, close monitoring and frequent reassessment are recommended, especially in patients managed conservatively or before definitive surgical drainage [11,18].

Overall, the available evidence supports a multimodal approach to airway assessment. Careful clinical examination, flexible endoscopic evaluation, and contrast-enhanced computed tomography provide complementary information and should all be incorporated into the initial evaluation. This comprehensive assessment not only identifies patients at increased risk of airway compromise but also facilitates selection of the most appropriate airway management strategy.

### 4.2. Endotracheal Intubation Versus Tracheostomy

Once the need for airway protection has been established, the next challenge is selecting the most appropriate technique. The choice between endotracheal intubation and tracheostomy remains one of the most debated aspects of airway management in patients with cervical infections [6,16]. Despite the growing number of published studies, no high-quality evidence clearly supports the superiority of one technique over the other [11]. Consequently, current practice is largely based on careful patient assessment, anticipated airway difficulty, disease severity, and the experience of the multidisciplinary team.

Historically, tracheostomy was considered the preferred airway management strategy in patients with advanced deep neck infections because it provided a definitive airway while avoiding manipulation of an inflamed upper airway [23,24]. Earlier reports recommended a low threshold for surgical airway, particularly in patients with Ludwig’s angina, extensive cellulitis, severe trismus, or rapidly progressive airway compromise.

However, contemporary difficult airway management has increasingly incorporated videolaryngoscopy, flexible bronchoscopic intubation, and structured difficult airway algorithms. In particular, videolaryngoscopy has assumed an increasingly prominent role in anticipated difficult airway management, providing improved glottic visualization and facilitating tracheal intubation in challenging anatomical conditions. Recent multidisciplinary recommendations support its broad implementation in both planned and emergency airway management [25]. Nevertheless, direct evidence regarding videolaryngoscopy specifically in patients with deep neck infections or acute infectious epiglottitis/supraglottitis remains limited, and recommendations derived from the broader difficult airway literature should therefore be applied cautiously to infection-related airway obstruction. Older techniques, such as retrograde intubation, are now rarely used in routine clinical practice and have largely been replaced by more reliable and less invasive advanced airway techniques [7,23].

Within the studies included in the present review, endotracheal intubation represented the most frequently adopted initial airway management strategy, particularly when performed by experienced anesthesiologists using advanced airway techniques [6,9,16]. Awake intubation, whenever feasible, offers the advantage of maintaining spontaneous ventilation and airway tone during airway instrumentation [7,26,27]. These techniques provide additional options for securing the airway in appropriately selected patients; however, their specific impact on the need for primary tracheostomy in cervical infections cannot be established from the available evidence. Successful intubation also remains highly dependent on operator experience and the immediate availability of alternative airway devices.

On the other hand, the reviewed literature consistently confirms that tracheostomy remains an essential option in selected patients. Extensive multispace infections, marked distortion of the upper airway, descending necrotizing mediastinitis, failure of endotracheal intubation, or the anticipated need for prolonged postoperative airway protection continue to represent the most frequent indications for surgical airway [6,10,17]. Importantly, the higher incidence of tracheostomy reported in patients with severe disease should not be interpreted as evidence of inferior outcomes associated with the procedure, but rather as a consequence of greater disease severity and more complex airway anatomy.

Apparently contrasting findings regarding tracheostomy were reported by Kim et al. [10] and Gehrke et al. [16]. In the small cohort by Kim et al. [10], early tracheostomy was performed in 4/20 patients and was associated with mortality; however, 5/20 patients died, with all deaths attributed to septic shock and multiorgan failure rather than airway-related causes. Conversely, Gehrke et al. [16] incorporated early tracheostomy into their treatment algorithm for patients with mediastinal involvement, among whom tracheostomy was performed in 82.2%, compared with 3.4% of patients without mediastinal involvement. These apparently divergent findings should be interpreted cautiously, as both studies were retrospective and the choice of airway strategy was strongly influenced by disease severity, raising the possibility of confounding by indication.

Another point that deserves consideration is postoperative airway management. While some centers routinely perform tracheostomy in patients with extensive cervical infections, others advocate prolonged postoperative endotracheal intubation followed by serial clinical and endoscopic reassessment [6,9]. Although both strategies have been reported with satisfactory outcomes, the available evidence remains insufficient to recommend one approach over the other. Instead, the decision should be individualized according to the expected resolution of airway edema, the extent of infection, the need for repeated surgical procedures, and the patient’s overall clinical condition.

Current difficult airway guidelines emphasize the importance of planning multiple airway strategies before induction of general anesthesia and ensuring immediate availability of a surgical airway whenever difficult intubation is anticipated [26,27]. This concept is particularly relevant in cervical infections, where airway conditions may deteriorate rapidly and repeated airway manipulation may increase edema, bleeding, and the risk of complete airway obstruction [11,16]. Therefore, close collaboration between anesthesiologists and otolaryngologists remains essential throughout airway management, allowing rapid transition from endotracheal intubation to tracheostomy whenever necessary.

Once the decision to secure the airway has been made, the choice of technique should be individualized according to the anticipated airway difficulty, the anatomical extent of infection, the expected duration of airway protection, and the expertise of the multidisciplinary team. Careful preoperative assessment, availability of advanced airway equipment, and early multidisciplinary planning appear to be key elements for safe airway management while minimizing procedure-related complications. Although specific management strategies have been proposed, including a therapeutic algorithm incorporating early tracheostomy in patients with mediastinal involvement [16] and a risk-stratification approach based on abscess location and laryngeal edema [20], these strategies derive from retrospective single-center studies and have not been externally validated. Therefore, the available evidence remains insufficient to support a universally applicable standardized decision algorithm.

### 4.3. Predictors of Airway Intervention

One of the most clinically relevant findings emerging from the present review is that the need for airway intervention can often be anticipated through the integration of clinical, radiological, and endoscopic findings [9,16,18]. Although individual studies identified different risk factors, a common observation was that no single variable accurately predicts airway deterioration. Instead, the decision to secure the airway should rely on a comprehensive assessment of the patient rather than on isolated clinical or imaging findings.

Among clinical variables, signs of progressive upper airway obstruction consistently represented the strongest indicators for airway intervention. Dyspnea, stridor, inability to manage oral secretions, dysphagia, muffled voice, and rapidly progressive cervical swelling were repeatedly associated with an increased likelihood of endotracheal intubation or tracheostomy [9,16,18]. In patients with acute epiglottitis or supraglottitis, stridor, dyspnea, sialorrhea, epiglottic abscess, and supraglottic extension of edema were associated with an increased likelihood of airway intervention [9,18,22]. These findings are consistent with the meta-analysis by Sideris et al. [5], which identified stridor (RR 7.15), epiglottic abscess (RR 2.45), and diabetes mellitus (RR 2.15) as significant predictors of airway intervention. Notably, diabetes did not emerge as a consistent predictor among the primary studies included in the present review. Similarly, Booth et al. [11] reported a pooled airway-intervention rate of 15.6% (95% CI 12.9–18.8) and a failed-intubation rate of 4.2%.

Radiological assessment plays an equally important role in risk stratification. Contrast-enhanced computed tomography not only confirms the diagnosis but also provides valuable information regarding the anatomical extent of infection [10,16,17]. The involvement of multiple deep neck spaces, particularly the parapharyngeal and retropharyngeal spaces, together with descending mediastinitis, was consistently associated with a higher frequency of airway intervention across the reviewed studies. These findings emphasize that imaging should be considered an integral component of airway evaluation rather than solely a tool for surgical planning [10,17].

Endoscopic examination further complements clinical and radiological assessment [9,18]. Flexible nasolaryngoscopy allows direct visualization of supraglottic edema, arytenoid involvement, airway narrowing, and secretion pooling, providing real-time information regarding the severity of airway compromise [22]. Likewise, postoperative endoscopic reassessment may facilitate decisions regarding extubation or the need for prolonged airway protection, particularly in patients with severe cervical infections [9,10].

Despite these advances, predicting airway deterioration remains challenging [11,16]. Most of the available evidence originates from retrospective single-center studies, and the proposed predictors have not been consistently validated across different populations. Furthermore, airway intervention is influenced not only by disease severity but also by institutional protocols, operator experience, and the availability of advanced airway techniques, making direct comparison between studies difficult.

Recently, increasing attention has been directed toward the use of artificial intelligence (AI) and machine-learning models to improve clinical risk prediction [28]. This pilot study included 392 patients; given that it was conducted at the same institution as Chen et al. [17] and covered an overlapping study period, some degree of cohort overlap cannot be excluded. By integrating multiple clinical, laboratory, endoscopic, and radiological variables, these approaches may overcome some of the limitations of traditional single-factor risk assessment and support earlier identification of patients at high risk for airway deterioration [28]. Although AI-based prediction models have shown promising preliminary results, current evidence remains limited, and prospective multicenter validation is required before these tools can be incorporated into routine clinical decision-making. At present, AI should therefore be regarded as a potential adjunct to, rather than a replacement for, comprehensive clinical assessment.

Overall, the available literature supports a multimodal approach to risk stratification in patients with cervical infections. Careful integration of clinical presentation, flexible endoscopy, and cross-sectional imaging remains the most reliable strategy for identifying patients who may require early airway intervention, while emerging AI-based predictive models represent a promising area for future research.

### 4.4. Clinical Implications

The findings of the present review have several practical implications for clinicians involved in the management of cervical infections [11,16]. An important clinical consideration emerging from the present review is the need for early recognition of progressive airway compromise. However, the included studies did not specifically evaluate the impact of timing of airway intervention on clinical outcomes. From a clinical perspective, progression to severe airway compromise may result in a more challenging scenario in which both endotracheal intubation and surgical airway become technically more demanding [11,26].

First, airway management should always be considered a dynamic process rather than a single therapeutic decision [9,18]. Patients who initially appear clinically stable may deteriorate rapidly because of progressive edema, abscess expansion, or extension of the infection into adjacent cervical spaces. Consequently, repeated airway assessment is as important as the initial evaluation, particularly during the first hours after hospital admission and following surgical drainage [11].

Second, the reviewed literature supports the use of a multidisciplinary approach involving anesthesiologists, otolaryngologists, maxillofacial surgeons, radiologists, and intensivists whenever appropriate [11,26]. Airway management decisions should be based on the integration of clinical findings, flexible nasolaryngoscopy, and contrast-enhanced computed tomography rather than on a single parameter [9,16,17]. This comprehensive evaluation not only facilitates early identification of patients at increased risk of airway deterioration but also assists in selecting the most appropriate airway management strategy.

Another important consideration is that complete airway obstruction represents a particularly challenging clinical scenario, potentially increasing the risk of failed intubation, hypoxemia, and the need for rescue surgical airway [6,14,18,26,27]. Conversely, unnecessary prophylactic airway intervention may expose patients to avoidable complications associated with prolonged intubation or tracheostomy [19,20]. The available evidence therefore supports individualized decision-making based on disease severity, anatomical extension, and anticipated airway difficulty rather than routine adoption of a single airway strategy [6,16].

Finally, advanced airway devices, standardized difficult airway algorithms, and emerging predictive models may provide additional tools to support individualized airway management [20,25,26,28]. However, their specific impact in patients with cervical infections requires further investigation. Future research should focus on validating reliable risk-stratification tools capable of identifying patients requiring early airway intervention while avoiding unnecessary invasive procedures. Validated risk-stratification tools may facilitate a more personalized approach to airway management and further improve patient safety.

### 4.5. Limitations

The present review has several limitations. Most included studies were retrospective single-center cohorts, making them prone to selection bias and limiting the generalizability of the findings. In addition, more than half of the overall study population was derived from a single nationwide administrative database study [19], which provided limited detail on airway decision-making and specific airway management techniques.

Moreover, the available literature is highly heterogeneous, encompassing different clinical entities, disease severity, and institutional airway management protocols, with no standardized criteria for endotracheal intubation or tracheostomy. Consequently, direct comparison between studies was challenging, and a quantitative meta-analysis was not feasible.

Another limitation is the lack of validated predictive models for airway intervention. Although several studies identified clinical, radiological, and endoscopic factors associated with airway compromise, these predictors have not been prospectively validated or incorporated into widely accepted decision-making tools.

In addition, the search strategy was primarily focused on identifying studies addressing airway intervention in cervical infections and did not include specific search terms for individual airway devices or techniques, such as videolaryngoscopy, GlideScope, C-MAC, awake intubation, or flexible bronchoscopic intubation. Consequently, the device-specific literature, particularly contemporary evidence regarding videolaryngoscopy, may have been underrepresented. The discussion of these techniques should therefore be interpreted as providing context on contemporary difficult airway practice rather than as evidence systematically identified by the present review.

Finally, only studies published in English were included; therefore, relevant evidence published in other languages may not have been captured.

### 4.6. Future Directions

Future prospective multicenter studies are necessary to establish standardized criteria and validate integrated prediction models for airway intervention. Importantly, the optimal timing of airway protection remains insufficiently investigated, as the available studies have not specifically evaluated time-to-intervention or stratified clinical outcomes according to timing. Prospective studies should therefore assess whether the timing of airway intervention independently influences airway-related complications and clinical outcomes. Emerging machine-learning approaches may further improve risk stratification and support clinical decision-making.

Despite these limitations, this review provides a comprehensive overview of current evidence on airway management in adult cervical infections, highlighting key factors influencing airway assessment, the choice of airway technique, and predictors of airway intervention.

## 5. Conclusions

Airway management in adult patients with cervical infections remains a complex clinical challenge that requires prompt recognition, accurate risk stratification, and close multidisciplinary collaboration.

Current evidence does not establish the superiority of endotracheal intubation or tracheostomy, and the choice of airway technique should therefore be individualized according to clinical presentation, anatomical findings, anticipated airway difficulty, and expected duration of airway protection.

Contemporary airway devices and advanced intubation techniques provide additional options for airway management; however, their specific impact in patients with cervical infections remains insufficiently characterized in the available literature. Tracheostomy remains an essential option in patients with extensive disease, severe airway distortion, or when prolonged airway protection is anticipated. Careful integration of clinical assessment, flexible nasolaryngoscopy, and contrast-enhanced computed tomography may help identify patients at increased risk of airway deterioration and guide timely intervention.

Future prospective multicenter studies are necessary to establish standardized criteria for airway intervention and to validate predictive models capable of supporting clinical decision-making. Until stronger evidence becomes available, airway management should be individualized, with treatment decisions based on multidisciplinary evaluation and the expertise of the treating team.

## Figures and Tables

**Table 1:** Characteristics of the included studies.

Study	Year	Country	Study Design	Disease/Condition	Patients (*n*)
Wolfe et al. [7]	2011	USA	Retrospective cohort	DNI and Ludwig’s angina	29
Garcia et al. [13]	2012	Portugal	Retrospective cohort	DNI admitted to ICU	54
Tapiovaara et al. [6]	2017	Finland	Retrospective cohort	DNI	202
Tapiovaara et al. [14]	2019	Finland	Retrospective cohort	Acute epiglottitis/supraglottitis	42
Shaikh et al. [15]	2020	Qatar	Retrospective cohort	Acute adult supraglottitis	118
Chen et al. [17]	2021	Taiwan	Retrospective cohort	DNI	403
Felton et al. [18]	2021	USA	Retrospective cohort	Acute adult epiglottitis	70
Pineau et al. [22]	2021	France	Retrospective cohort	Acute adult epiglottitis	28
Gehrke et al. [16]	2022	Germany	Retrospective cohort	DNI with/without mediastinitis	218
Penella et al. [9]	2022	Spain	Retrospective cohort	Acute infectious supraglottitis	88
Kim et al. [10]	2022	South Korea	Retrospective cohort	Descending necrotizing mediastinitis secondary to DNI	20
Konishi et al. [19]	2023	Japan	Nationwide retrospective database study	Parapharyngeal and retropharyngeal abscesses	1882
Iwata et al. [20]	2025	Japan	Retrospective case–control study	Severe odontogenic DNI	64
Hennocq et al. [21]	2026	France	Prospective cohort plus retrospective cohort	Cervicofacial cellulitis	79

Abbreviations: DNI: deep neck infection; ICU: intensive care unit; *n*: number.

**Table 2:** Objectives, key airway-related findings and limitations of the included studies.

Study	Objective	Key Airway-Related Findings	Main Limitations
Wolfe et al. [7]	To determine whether surgical airway is necessary in patients with deep neck infections and Ludwig’s angina.	Advanced airway techniques, including fiberoptic, GlideScope, and retrograde intubation, allowed airway control without surgical airway in the reported cohort.	Small retrospective single-center series; highly dependent on local expertise in advanced airway management.
Garcia et al. [13]	To identify predictors of severe infection and complicated clinical course in surgically drained DNI patients admitted to ICU.	Retropharyngeal involvement was associated with severe infection, while parapharyngeal involvement was associated with a complicated course including reintubation or tracheostomy.	ICU-only population
Tapiovaara et al. [6]	To compare intubation and tracheostomy in patients with DNI.	Intubation was the most common airway strategy and immediate extubation after surgery was frequently possible; tracheostomy was associated with longer hospitalization and more severe disease.	Retrospective design; no standardized decision algorithm for airway management.
Tapiovaara et al. [14]	To compare intubation and tracheostomy in adult acute epiglottitis/supraglottitis.	Intubation and tracheostomy were both used to secure the airway; tracheostomy was less costly but showed a trend toward more complications and longer sick leave.	Small sample size; only patients requiring airway intervention were included.
Shaikh et al. [15]	To describe presentation, diagnosis, and management of acute adult supraglottitis.	A substantial proportion of patients required airway intervention; failed intubation and tracheostomy were reported, supporting the need for early airway preparedness.	Retrospective single-center ICU cohort
Gehrke et al. [16]	To analyze treatment and outcomes of DNI with and without mediastinal involvement and propose a therapeutic algorithm.	Patients with mediastinal involvement had markedly higher tracheostomy rates; early tracheostomy was considered beneficial within their treatment algorithm.	Retrospective single-center study; airway strategy linked to institutional surgical protocol.
Chen et al. [17]	To identify factors associated with the need for tracheostomy in DNI.	Age ≥ 65 years, involvement of ≥3 deep neck spaces, and mediastinitis were independent predictors of tracheostomy.	Retrospective single-center cohort; no external validation of predictors.
Felton et al. [18]	To characterize adult epiglottitis and identify predictors of airway intervention.	Most patients did not require intervention, but stridor, dyspnea and voice changes were associated with need for advanced airway management; severe adverse airway events were reported.	Limited sample size; retrospective chart review.
Pineau et al. [22]	To identify clinical and endoscopic criteria associated with the decision to intubate adults with acute epiglottitis.	Dyspnea and supraglottic extension of edema were the main factors associated with the decision to intubate.	Small retrospective single-center cohort.
Penella et al. [9]	To evaluate epidemiology, management, outcomes, and predictors of airway intervention in adult infectious supraglottitis.	Airway intervention was required in 17% of patients; epiglottic abscess, sialorrhea, and smoking were associated with airway intervention.	Retrospective single-center study; limited number of airway events.
Kim et al. [10]	To determine optimal airway management in descending necrotizing mediastinitis secondary to DNI.	Short-term orotracheal intubation after initial surgery was safe in selected patients; upfront tracheostomy was not routinely supported.	Very small cohort; rare disease; retrospective design.
Konishi et al. [19]	To describe treatment patterns and outcomes in adults undergoing emergency surgery for parapharyngeal or retropharyngeal abscesses.	Parapharyngeal abscesses were associated with more intensive treatment, including higher use of tracheostomy and ICU admission, and worse outcomes than retropharyngeal abscesses.	Administrative database; limited detail on airway decision-making and technique.
Iwata et al. [20]	To identify predictors of difficult postoperative airway management in severe odontogenic DNI.	Arytenoid edema, laryngeal edema, and para-/retropharyngeal abscesses were associated with prolonged intubation or tracheostomy.	Small retrospective cohort; focused on postoperative airway management only.
Hennocq et al. [21]	To evaluate trismus reversibility after induction and identify factors associated with tracheostomy in cervicofacial cellulitis.	Trismus improved after induction in 17/17 patients; dysphagia and non-homogeneous collections were associated with tracheostomy.	Single-center study; limited prospective airway measurements.

Abbreviations: DNI: deep neck infection; ICU: intensive care unit.

**Table 3:** Airway management strategies and airway-related outcomes.

Study	Airway Strategy Evaluated	Airway-Related Outcome	Reported Predictors or Risk Features
Wolfe et al. [7]	Advanced intubation techniques versus surgical airway	No patient required surgical airway; advanced airway techniques were used successfully in selected patients.	Airway compromise defined by secretion management, anxiety, stridor, trismus, dysphonia, or dysphagia.
Garcia et al. [13]	Airway support in ICU patients with DNI	Complicated course included reintubation and tracheostomy.	Retropharyngeal location (52% vs. 7%, *p* < 0.001) and multiple-space involvement (52% vs. 13%, *p* = 0.002) were associated with severe infection; parapharyngeal location (60% vs. 8%, *p* < 0.001) was associated with a complicated course.
Tapiovaara et al. [6]	Intubation versus primary/secondary tracheostomy	Intubation was the most frequent airway strategy; tracheostomy was associated with longer hospitalization and a more severe clinical course.	Submental space involvement was associated with primary tracheostomy (11/32, 34%; *p* = 0.005); mediastinal extension was associated with prolonged tracheostomy dependence (*p* = 0.007) and hospitalization (*p* < 0.001).
Tapiovaara et al. [14]	Intubation versus tracheostomy in acute epiglottitis/supraglottitis	Both methods secured the airway; tracheostomy was less costly but tended toward more complications and longer recovery.	Not specifically assessed; only patients requiring airway intervention were included.
Shaikh et al. [15]	Endotracheal intubation and tracheostomy	Endotracheal intubation was performed in 54/118 patients (45.8%); intubation was unsuccessful in 12/118 (10.2%), and tracheostomy was performed in 15/118 (12.7%).	Dysphagia and fever were associated with progression to Ludwig’s angina; patients developing Ludwig’s angina had a higher frequency of airway intervention.
Gehrke et al. [16]	Early tracheostomy within DNI treatment algorithm	Tracheostomy was performed in 82.2% of patients with mediastinal involvement versus 3.4% without mediastinal involvement.	Mediastinal involvement was strongly associated with tracheostomy (OR 128.7, 95% CI 42.12–393.4).
Chen et al. [17]	Tracheostomy in DNI	Tracheostomy was required in 44/403 patients.	Age ≥ 65 years (aOR 2.45, 95% CI 1.16–5.16), ≥3 involved spaces (aOR 4.49, 95% CI 2.15–9.36), and mediastinitis (aOR 14.80, 95% CI 5.10–42.97).
Felton et al. [18]	Intubation, cricothyrotomy, or tracheostomy	Airway intervention was required in 17.1%; emergent surgical airway and severe airway-related morbidity/mortality occurred.	Stridor (70.0% vs. 2.0%, *p* < 0.001), voice alteration (75.0% vs. 31.0%, *p* = 0.008), and dyspnea (41.7% vs. 3.4%, *p* = 0.001) were associated with airway intervention.
Pineau et al. [22]	Intubation versus surveillance	Intubation was required in 10/28 patients.	Dyspnea (OR 50.6; 95% CI 2.7–940.1) and supraglottic extension of edema (OR 42.2; 95% CI 2.2–799.5) were the main factors associated with the decision to intubate.
Penella et al. [9]	Conservative treatment versus airway intervention	Airway intervention was required in 15/88 patients: 9 intubations and 6 tracheostomies.	Epiglottic abscess (aOR 8.31, 95% CI 1.65–53.40), sialorrhea (aOR 5.69, 95% CI 1.24–28.70), and smoking (aOR 5.15, 95% CI 1.24–28.80).
Kim et al. [10]	Short-term orotracheal intubation versus early/late tracheostomy	Early tracheostomy (4/20) was associated with mortality; 5/20 patients died from septic shock/multiorgan failure, not airway-related causes.	No specific predictors of airway intervention were identified.
Konishi et al. [19]	Tracheostomy as part of treatment course	Tracheostomy was more frequent in parapharyngeal abscesses than in retropharyngeal abscesses.	Parapharyngeal abscess was associated with a higher tracheostomy rate than retropharyngeal abscess (36% vs. 25%, *p* < 0.001).
Iwata et al. [20]	Short-term intubation versus prolonged intubation/tracheostomy	Difficult postoperative airway management occurred in 7/64 patients.	Arytenoid edema, laryngeal edema, retropharyngeal abscess, parapharyngeal abscess, higher inflammatory markers.
Hennocq et al. [21]	Intubation after induction and tracheostomy	Mouth opening increased after induction in 17/17 measured patients; dysphagia and non-homogeneous collections were associated with tracheostomy.	Dysphagia (OR 6.89, 95% CI 1.56–58.24) and non-homogeneous collection; homogeneous collection was protective (OR 0.20, 95% CI 0.03–0.78).

Abbreviations: DNI: deep neck infection; ICU: intensive care unit; OR: odds ratio; aOR: adjusted odds ratio; CI: confidence interval.

## Data Availability

No new data were created or analyzed in this study. Data sharing is not applicable to this article.

## References

[1] Wang L.F., Kuo W.R., Tsai S.M., Huang K.J. (2003). Characterizations of Life-Threatening Deep Cervical Space Infections: A Review of One Hundred Ninety-Six Cases. Am. J. Otolaryngol..

[2] Hanna J., Brauer P.R., Berson E., Mehra S. (2019). Adult Epiglottitis: Trends and Predictors of Mortality in Over 30 Thousand Cases from 2007 to 2014. Laryngoscope.

[3] Prado-Calleros H.M., Jiménez-Fuentes E., Jiménez-Escobar I. (2016). Descending Necrotizing Mediastinitis: Systematic Review on Its Treatment in the Last 6 Years, 75 Years after Its Description. Head Neck.

[4] Estrera A.S., Landay M.J., Grisham J.M., Sinn D.P., Platt M.R. (1983). Descending Necrotizing Mediastinitis. Surg. Gynecol. Obstet..

[5] Sideris A., Holmes T.R., Cumming B., Havas T. (2020). A Systematic Review and Meta-Analysis of Predictors of Airway Intervention in Adult Epiglottitis. Laryngoscope.

[6] Tapiovaara L., Bäck L., Aro K. (2017). Comparison of Intubation and Tracheotomy in Patients with Deep Neck Infection. Eur. Arch. Otorhinolaryngol..

[7] Wolfe M.M., Davis J.W., Parks S.N. (2011). Is Surgical Airway Necessary for Airway Management in Deep Neck Infections and Ludwig Angina?. J. Crit. Care.

[8] Potter J.K., Herford A.S., Ellis E. (2002). Tracheotomy versus Endotracheal Intubation for Airway Management in Deep Neck Space Infections. J. Oral Maxillofac. Surg..

[9] Penella A., Mesalles-Ruiz M., Portillo A., Huguet Llull G., Bagudà E., Capelleras M., Nogués J., Mañós M., Gonzàlez-Compta X. (2022). Acute Infectious Supraglottitis in Adult Population: Epidemiology, Management, Outcomes and Predictors of Airway Intervention. Eur. Arch. Otorhinolaryngol..

[10] Kim Y., Han S., Cho D.G., Jung W.S., Cho J.H. (2022). Optimal Airway Management in the Treatment of Descending Necrotizing Mediastinitis Secondary to Deep Neck Infection. J. Oral Maxillofac. Surg..

[11] Booth A.W.G., Pungsornruk K., Llewellyn S., Sturgess D., Vidhani K. (2024). Airway Management of Adult Epiglottitis: A Systematic Review and Meta-Analysis. BJA Open.

[12] Tricco A.C., Lillie E., Zarin W., O’Brien K.K., Colquhoun H., Levac D., Moher D., Peters M.D.J., Horsley T., Weeks L. (2018). PRISMA Extension for Scoping Reviews (PRISMA-ScR): Checklist and Explanation. Ann. Intern. Med..

[13] Garcia T., Rios M., Paiva J.A. (2012). Predictors of Severity in Deep Neck Infections Admitted to the Intensive Care Unit. Anaesth. Intensive Care.

[14] Tapiovaara L.K., Aro K.L.S., Bäck L.J.J., Koskinen A.I.M. (2019). Comparison of Intubation and Tracheotomy in Adult Patients with Acute Epiglottitis or Supraglottitis. Eur. Arch. Otorhinolaryngol..

[15] Shaikh N., Nawaz S., Ahmad K., Al Maslamani M. (2020). Acute Adult Supraglottitis: An Impending Threat to Patency of Airway and Life. Cureus.

[16] Gehrke T., Scherzad A., Hagen R., Hackenberg S. (2022). Deep Neck Infections with and without Mediastinal Involvement: Treatment and Outcome in 218 Patients. Eur. Arch. Otorhinolaryngol..

[17] Chen S.L., Young C.K., Tsai T.Y., Chien H.T., Kang C.J., Liao C.T., Huang S.F. (2021). Factors Affecting the Necessity of Tracheostomy in Patients with Deep Neck Infection. Diagnostics.

[18] Felton P., Lutfy-Clayton L., Smith L.G., Visintainer P., Rathlev N.K. (2021). A Retrospective Cohort Study of Acute Epiglottitis in Adults. West. J. Emerg. Med..

[19] Konishi T., Sakata A., Inokuchi H., Kumazawa R., Matsui H., Fushimi K., Tanabe M., Seto Y., Yasunaga H. (2023). Treatments and Outcomes of Adult Parapharyngeal and Retropharyngeal Abscess: 1882 Cases from a Japanese Nationwide Database. Am. J. Otolaryngol..

[20] Iwata E., Inokuchi G., Kawakami M., Matsui T., Kusumoto J., Tachibana A., Akashi M. (2025). Predictive Factors in Difficult Postoperative Airway Management of Severe Odontogenic Deep Neck Infection. Odontology.

[21] Hennocq Q., Plantin S., Salettes R., Bouaoud J., Raux M., Bertolus C., Foy J.P. (2026). Prognostic Factors for Tracheostomy in Patients with Cervicofacial Cellulitis. J. Stomatol. Oral Maxillofac. Surg..

[22] Pineau P.M., Gautier J., Pineau A., Emam N., Laccourreye L., Boucher S. (2021). Intubation decision criteria in adult epiglottitis. Eur. Ann. Otorhinolaryngol. Head Neck Dis..

[23] Ovassapian A., Tuncbilek M., Weitzel E.K., Joshi C.W. (2005). Airway Management in Adult Patients with Deep Neck Infections: A Case Series and Review of the Literature. Anesth. Analg..

[24] Moreland L.W., Corey J., McKenzie R. (1988). Ludwig’s Angina: Report of a Case and Review of the Literature. Arch. Intern. Med..

[25] Gómez-Ríos M.Á., Van Zundert A.A.J., McNarry A.F., Law J.A., Higgs A., De Jong A., Jaber S., Karamchandani K., Hansel J., Saracoglu K.T. (2025). Guidelines on Strategies for the Universal Implementation of Videolaryngoscopy. Eur. J. Anaesthesiol..

[26] Apfelbaum J.L., Hagberg C.A., Connis R.T., Abdelmalak B.B., Agarkar M., Dutton R.P., Fiadjoe J.E., Greif R., Klock P.A., Mercier D. (2022). American Society of Anesthesiologists Practice Guidelines for Management of the Difficult Airway. Anesthesiology.

[27] Ahmad I., El-Boghdadly K., Bhagrath R., Hodzovic I., McNarry A.F., Mir F., O’Sullivan E.P., Patel A., Stacey M., Vaughan D. (2020). Difficult Airway Society Guidelines for Awake Tracheal Intubation (ATI) in Adults. Anaesthesia.

[28] Chen S.L., Chin S.C., Ho C.Y. (2022). Deep Learning Artificial Intelligence to Predict the Need for Tracheostomy in Patients of Deep Neck Infection Based on Clinical and Computed Tomography Findings—Preliminary Data and a Pilot Study. Diagnostics.

